# Paneth cells in farm animals: current status and future direction

**DOI:** 10.1186/s40104-023-00905-5

**Published:** 2023-08-15

**Authors:** Chenbin Cui, Lindeng Li, Lin Wu, Xinru Wang, Yao Zheng, Fangke Wang, Hongkui Wei, Jian Peng

**Affiliations:** 1 Department of Animal Nutrition and Feed Science, College of Animal Science and Technology, Huazhong Agricultural University, Wuhan, 430070 China; 2 The Cooperative Innovation Center for Sustainable Pig Production, Wuhan, 400700 China

**Keywords:** Antimicrobial peptide, Farm animal, Intestinal organoid, Intestine, Paneth cell

## Abstract

A healthy intestine plays an important role in the growth and development of farm animals. In small intestine, Paneth cells are well known for their regulation of intestinal microbiota and intestinal stem cells (ISCs). Although there has been a lot of studies and reviews on human and murine Paneth cells under intestinal homeostasis or disorders, little is known about Paneth cells in farm animals. Most farm animals possess Paneth cells in their small intestine, as identified by various staining methods, and Paneth cells of various livestock species exhibit noticeable differences in cell shape, granule number, and intestinal distribution. Paneth cells in farm animals and their antimicrobial peptides (AMPs) are susceptible to multiple factors such as dietary nutrients and intestinal infection. Thus, the comprehensive understanding of Paneth cells in different livestock species will contribute to the improvement of intestinal health. This review first summarizes the current status of Paneth cells in pig, cattle, sheep, horse, chicken and rabbit, and points out future directions for the investigation of Paneth cells in the reviewed animals.

## Introduction

Intestinal health is a popular topic and has attracted increasing attention in husbandry [1]. Improvements of growth performance and health status of livestock are closely related to intestine functions [2]. A healthy intestine is of vital importance for the digestion and absorption of dietary nutrients. As one of the most important immune organs, intestine possesses more than 70% of bodily immune cells and immunoglobulin A (IgA) secreted in the intestine [3]. Notably, the intestine is a complex organ, and the size and digestion function of the intestine change rapidly after birth [4]. The importance of the intestine in systemic health has been widely confirmed, for example, it can affect other organs such as liver (intestine-liver axis) and brain (intestine-brain axis) through microbiota-derived metabolites or bile acid [5, 6]. Therefore, maintaining the intestinal health of farm animals is the key to the increase in economic benefits in husbandry.

The intestinal epithelium consisting of a single layer of epithelial cells plays an important role in absorbing the digested dietary nutrients and separating the large amounts of microorganisms in the intestinal lumens from the lamina propria of intestine [7]. In addition to absorptive cells, many types of secretory cells exist in the intestinal epithelium, mainly including Paneth cells, goblet cells, and enteroendocrine cells, all of which are differentiated from intestinal stem cells (ISCs) [8]. Paneth cells was first identified by Gustav Schwalbe who found the presence of granule-containing cells in the crypts of human small intestine [9], and 5 years later, Josef Paneth designated these cells as Paneth cells [10]. As a type of long-lived epithelial cells, Paneth cells possess approximately one-month lifespan in small intestine [11]. In addition to immune cells such as macrophages and dendritic cells, Paneth cells are considered as an essential part of intestinal innate immunity due to their multiple functions such as antimicrobial peptide (AMP) secretion and ISC support [11]. Since Paneth cell impairment leads to severe human diseases such as Crohn's disease (CD) and necrotizing enterocolitis (NEC), the basic studies of Paneth cells have been mainly conducted in humans and mice [12–14]. However, Paneth cells in the small intestine of livestock, especially their biological functions, are poorly understood. To promote a comprehensive understanding of Paneth cells in farm animals, this review summarizes the current knowledge of Paneth cells in farm animals such as pig, cattle and sheep, and proposes the future directions for research on Paneth cells in the reviewed animals.

## Brief retrospect on Paneth cells in small intestine

**Fig. 1:**
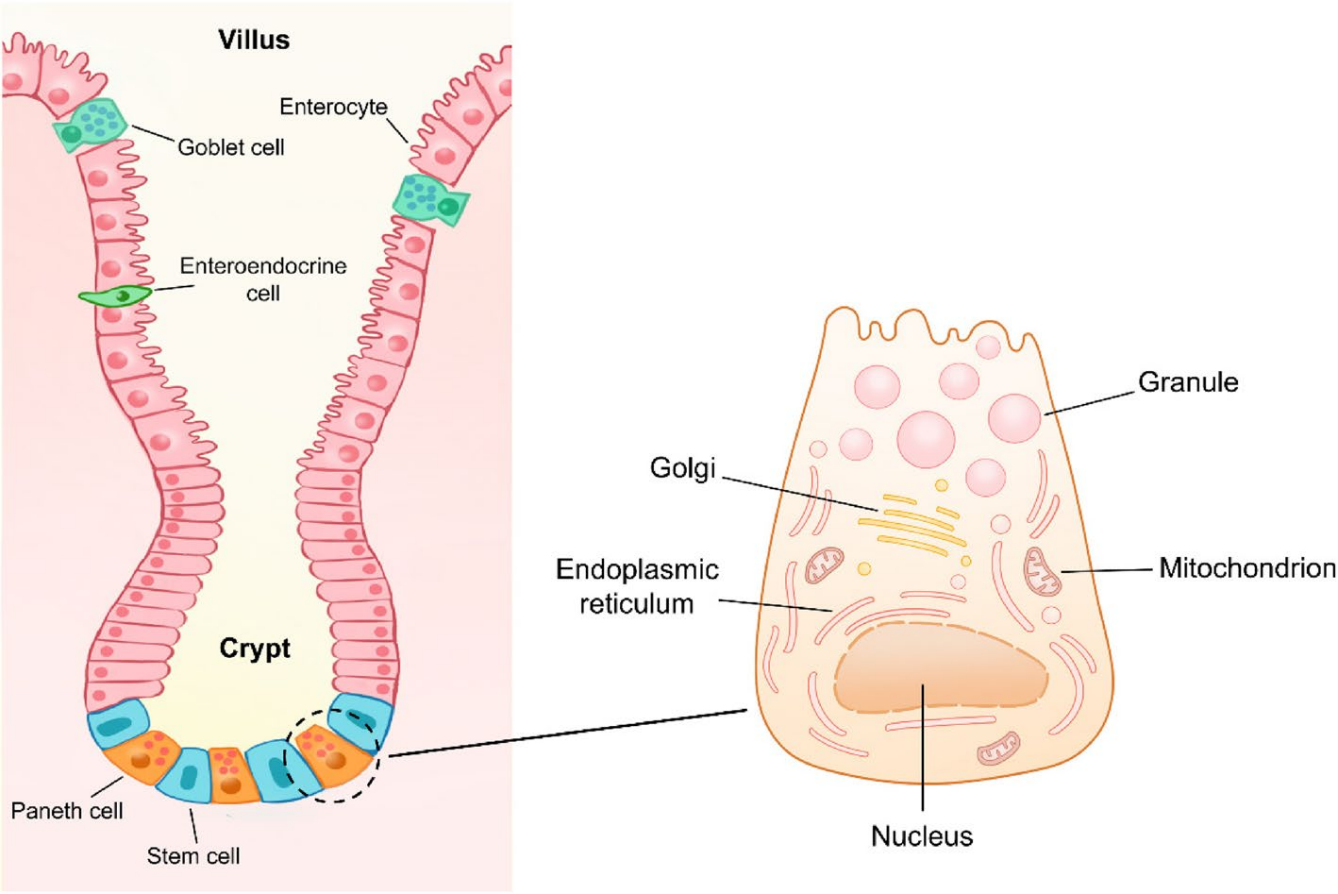
The location and structure of Paneth cells. Paneth cells are located at the bottom of small intestinal crypts and adjacent to intestinal stem cells. Paneth cells contain abundant endoplasmic reticulum and Golgi network to perform the continuous secretion of granules

The intestinal epithelium is structurally composed of villus and crypts, and villus length and crypt depth are generally used to measure intestinal injury [15, 16]. In contrast to the location of other secretory cells on villus, Paneth cells reside in the bottom of the crypts and are adjacent to ISCs (Fig. 1).

**Fig. 2:**
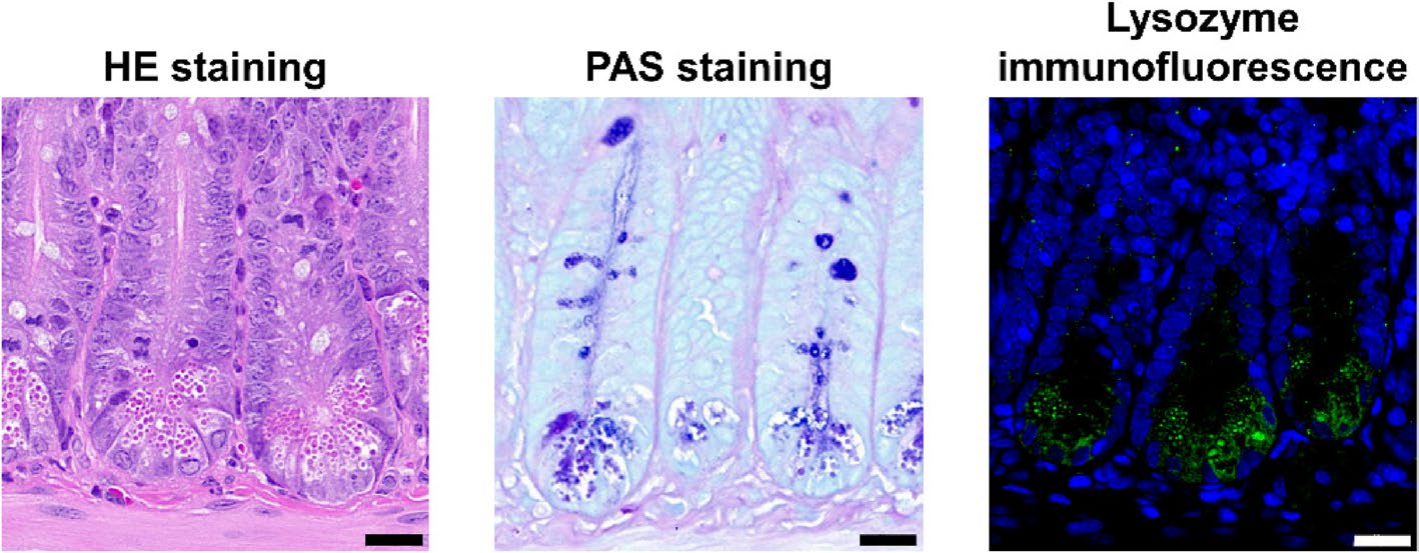
The secretory granules in murine Paneth cells can be labelled by multiple staining methods such as HE staining (left), PAS staining (middle), and lysozyme staining (right). Scale bar: 20 μm

As a type of columnar epithelial cells in small intestine, human or murine Paneth cells possess structural specificity, compared with other epithelial cells. The most striking feature of Paneth cells is the presence of abundant large granules in apical cytoplasm [11]. These granules can be visualized via various methods such as hematoxylin–eosin (HE) staining, periodic acid-Schiff (PAS) staining, and lysozyme immunofluorescence (IF) (Fig. 2). Due to the continuous secretory nature, Paneth cells have abundant endoplasmic reticulum, trans-Golgi network, and mitochondria to synthesize proteins and supply energy (Fig. 1) [17].

Paneth cell granules contain various AMPs such as α-defensin and lysozyme [18]. Abundant AMPs could be released into the intestinal lumen after multiple stimulations such as bacteria and carbamyl choline [19]. Paneth cell-derived AMPs contribute to maintain the healthy composition of intestinal microbiota and protect from bacterial infection [11]. These AMPs display strong bactericidal ability due to their properties of bacterial binding and membrane perforation [18]. Paneth cell-disrupted mice exhibit significant disorders in intestinal microbiota, and they are more susceptible to *Klebsiella* infection [20]. Considering the neighboring distribution of Paneth cells and ISCs, Paneth cells support the ISC niche by providing growth-promoting factors (such as Wnt3a and Dll4) and metabolites (such as lactate and cyclic ADP ribose) [21, 22]. In addition, Paneth cells can secrete cell apoptosis-triggering CD95 ligand, thus promoting the renewal of intestinal epithelium [23]. Recent study has demonstrated that Paneth cells act as phagocytes to remove apoptotic cells in crypts [24]. Paneth cell functions are comprehensively discussed in our review published recently [18].

## Paneth cells in various farm animal species

Paneth cells have been confirmed to be the guardians of small intestine in humans and mice [25]. However, less attention has been paid to Paneth cells in small intestine of farm animals. Considering that Paneth cells may also contribute to the maintenance of intestinal health, it is necessary to summarize the reports involving Paneth cells in different livestock species.

### Paneth cells in pig

In the last few decades, the presence of Paneth cells in the small intestine of pigs has been in controversy [26, 27], which results from the inconsistent staining findings of porcine Paneth cells. In 1982, Myer [28] reported that Paneth cells existed in the jejunal crypts of 5-month-old pigs via the staining of Mallory's phosphotungstic acid haematoxylin and phloxine tartrazine (PT). However, the Paneth cell granule size of pigs is relatively small compared with that of humans or mice, and the occurrence of Paneth cell granules is quite infrequent in the small intestinal crypts of pigs, which could be the main reasons for the controversy over the existence of Paneth cells in pigs [28]. This finding corrects the misconception that Paneth cells are absent in the porcine crypts. In 2005, Obremski et al. also identified acidophilic granules in the small intestinal crypts of 4-month-old gilts via HE staining, suggesting the presence of Paneth cells in gilts [29]. However, there is only no more than one Paneth cell per crypt, which is consistent with the report by Myer.

From 2013 to now, the efforts have been continuously made to explore whether Paneth cells are present in pigs or not. Two studies have reported that HE staining, PT staining, and toluidine blue (TB) staining fail to visualize Paneth cells in the small intestine of 0-, 6-, 28-day-old and 6–8-week-old pigs [30, 31]. In contrast, PT staining and TB staining successfully visualize the acidophilic granules in the ileal crypts of 21-day-old and 5-month-old pigs [32, 33]. These contradictory reports may be attributed to the small granule size and infrequent occurrence of porcine Paneth cells, as mentioned above. Furthermore, lysozyme, a Paneth cell-derived AMP, is a widely used Paneth cell marker in humans and mice [18]. Lysozyme staining to identify porcine Paneth cells also exhibits inconsistent results. Two studies have reported that there is no lysozyme positive cell in the small intestinal crypts of 6–8-week-old and 6-month-old pigs [30, 34]. On the contrary, lysozyme immunohistochemistry (IHC) and IF on pig jejunal and ileal sections have revealed that lysozyme positive Paneth cells are located in the intestinal crypts of 28-day-old and 5-month-old pigs [33, 35]. In addition, three dimensional intestinal organoids of livestock have been developed to be a promising tool for studies on intestinal epithelium [36]. Lysozyme staining display the presence of lysozyme positive cells in porcine intestinal organoids and organoid-derived monolayers, and the gene expression of *Lyz* encoding lysozyme is continuously upregulated in monolayers from d 1 to 3 after inoculating organoid-derived round cell cluster into 96-well plates [37]. The different levels of cross-reactivity of lysozyme antibodies may result in this discrepancy.

Another viewpoint to explain this discrepancy is that there could be a group of Paneth-like cells in the porcine small intestine. This is based on two reports that transmission electron microscopy (TEM) has shown that there are a group of large pyramid-shaped cells adjacent to ISCs in porcine ileal crypts, and these Paneth-like cells possess large supranuclear clear mucoid vesicles and small electron dense bodies in the apical cytoplasm, which are not entirely consistent with the morphology of human or murine Paneth cells [30, 33]. In addition, immunostaining results of porcine small intestinal crypts also support this viewpoint. Sex determining region Y-box 9 (Sox9) is required for Paneth cell differentiation and intestinal cell proliferation, and proliferative cells in crypts can be labeled by proliferating cell nuclear antigen (PCNA) [18]. In the bottom of porcine duodenum and jejunum, a group of Sox9^+^PCNA^−^ cells are observed through IHC, whose label is consistent with the label pattern of human and murine Paneth cells [31]. In mice, colonic c-Kit^+^Muc2^+^ cells may represent an counterpart analogous to the Paneth cells in small intestine [38]. Gonzalez et al. have reported that large amounts of Muc2^+^ cells are observed in porcine small intestinal crypts, suggesting the potential existence of c-Kit^+^Muc2^+^ cells [30]. However, this study is unable to find a proper porcine c-Kit antibody, thus failing to demonstrate the hypothesis that a Paneth cell counterpart (c-Kit^+^Muc2^+^ cell) may be present in porcine small intestinal crypts.

Single-cell mRNA sequencing (scRNA-seq) has been widely utilized to broaden the understanding of the cell composition of numerous organs [39]. A recent study using scRNA-seq has revealed that Paneth cells are absent in the ileal epithelium of three 6-month-old pigs, and in this study, the identification of porcine Paneth cells is based on the detection of Paneth cell markers *Lyz* and *Mmp7* [34]. However, another study of porcine ileal epithelium using scRNA-seq has demonstrated that the abundance of Paneth cells is increased slightly from d 0 to 1, and then continuously reduced until d 21 after birth [40]. The abundance of porcine ileal Paneth cells is extremely low (0.12%) at d 21 after birth, which may be responsible for the failure to detect ileal Paneth cells from 6-month-old pigs [34, 40].

Similar to Paneth cells in humans and mice, porcine Paneth cells are susceptible to dietary nutrients, toxins, and microorganisms. Compared with pigs fed with soybean hull diet, pigs fed with wheat straw diet exhibit the increased gene expression of *Lyz* in the ileum [41]. A diet supplemented with epidermal growth factor (EGF) promotes the expression of genes related to Paneth cell differentiation and increases lysozyme level in porcine jejunum during the post-weaning period, partly through EGF receptor signaling and Wnt/β-catenin signaling [42]. However, dietary nucleotide or antibiotic supplementation dramatically reduces porcine ileal Paneth cell area (which is calculated as length multiplied by width of Paneth cell-containing region) [32]. These studies highlight the importance of a proper diet in porcine Paneth cell development. Pig feeds are usually contaminated by deoxynivalenol (DON, also known as vomitoxin) [43]. DON challenge in piglets impairs porcine β-defensin expression that is correlated with the average daily gain (ADG) and the average daily feed intake (ADFI) of piglets [44]. For various viral infections, the responses of porcine Paneth cells are different. The *Lyz* expression in porcine intestinal organoid-derived monolayer is elevated after porcine deltacoronavirus (PDCoV) infection [37]. PDCoV has tendency to infect porcine stem/progenitor cells and enterocytes, suggesting that the upregulation of *Lyz* expression could be a protective reaction of host cells to resist infection [37]. Porcine epidemic diarrhea virus (PEDV) infection leads to a decrease in *Lyz* expression in porcine ileum rather than jejunum. PEDV can lower host pro-inflammatory cytokine expression to evade immune responses [45, 46]. Cell-extrinsic signaling such as interleukin (IL)-17 and IL-22 is able to simulate lysozyme production in Paneth cells [47, 48]. The difference in *Lyz* expression between porcine jejunum and ileum is attributed to the possibility that PEDV can inhibit the pro-inflammatory cytokine expression of immune cells in Peyer’s patch mainly located at ileum [49]. Similarly, in transmissible gastroenteritis virus (TGEV) infection, porcine Paneth cell number is significantly reduced [50]. Subsequent TGEV infection in IPEC-J2 cells shows that TGEV infection aggravates cell apoptosis, inhibits cell proliferation, and induces mitochondrium damage and reactive oxygen species (ROS) production in CD24^+^SSC^high^ cells (Paneth cells) [50]. Notably, the level of Dll4 (a Paneth cell-derived ISC-supporting factor) is downregulated in PEDV-infected porcine jejunum and IPEC-J2 cells, which could be the reason for the impaired ISC niche [50].

In summary, Paneth cells are present in porcine small intestine in spite of their infrequency. The scRNA-seq results suggest that the study of porcine Paneth cells should be conducted in young piglets. In addition, it is worth exploring that whether Paneth cells differ between sexes in pigs.

### Paneth cells in cattle

So far, there has been no in vivo study reporting the existence of Paneth cells in bovine small intestine. Bovine small intestinal organoids are successfully established from isolated bovine jejunal or ileal crypts, and IF staining reveals that bovine small intestinal organoids contain goblet cells and enteroendocrine cells (Paneth cell detection has not been conducted) [51, 52]. Another study using TEM has reported that Paneth cells containing few dense cytoplasmic vesicles are occasionally present in the crypt region of bovine ileal organoids [53]. However, the shape of bovine Paneth cells is irregular and the location of vesicles is scattered [53]. Subsequent mRNA-seq analysis has demonstrated that the genes related to Paneth cell differentiation (*Atoh1* and *Sox9*) and Paneth cell markers (*CD24* and *Lyz1*) are expressed in bovine ileal organoids, which further confirms the presence of Paneth cells in bovine small intestine [53]. Human and murine Paneth cells express abundant α-defensins responsible for killing pathogenic bacteria, whereas the bovine genome does not contain α-defensin genes. In bovine intestinal organoids, the expression of β-defensin genes (*Defb* and *Defb1*) is detected, and these two genes are considered as bovine Paneth cell markers [53]. Actually, bovine β-defensins were investigated long time ago. The level of β-defensins in the ileum of adult cattle is higher than that of fetal cattle. Notably, bovine colon exhibits higher β-defensin level than bovine ileum, suggesting the possibilities that Paneth cells might exist in bovine colon, or that there are a group of cells expressing β-defensin in bovine colon [54]. In addition, Luenser and Ludwig have reported the conservation of bovine defensins after comparing the amino-acid sequences between bovine defensins and human or mouse defensins [55].

In addition to canonical AMPs, bovine Paneth cells have also been found to possess other proteins such as lipopolysaccharide (LPS)-binding protein (LBP) and intelectin 2 (ITLN2). Murine Paneth cells have been identified as the main source of LBP [56]. LBP promotes LPS monomerization and present it to CD14, which highlights the important role of LBP in intestinal innate immunity [57]. LBP expression in bovine small intestine is higher than that in bovine colon [58], suggesting that bovine LBP may be also produced by Paneth cells, which remains to be further verified. ITLN2 is mainly derived from Paneth cells and goblet cells in mice [59, 60]. ITLN2 protein level is increased in Paneth cells and goblet cells within the ileocecal valve crypts of cattle treated with *Mycobacterium avium* subsp. *Paratuberculosis*, a Gram-positive bacterium causing paratuberculosis (PTB) [61, 62]. Importantly, receiver operating characteristic (ROC) analysis has revealed that bovine ITLN2 level can be used as a biomarker of PTB disease progression [61].

In conclusion, although there is no direct staining evidence demonstrating the existence of bovine Paneth cells in vivo, the intestinal organoid and mRNA-seq results indicate the presence of bovine Paneth cells. The infrequent occurrence of bovine Paneth cells and few secretory granules may be the reasons for failure to observe bovine Paneth cells in vivo.

### Paneth cells in sheep

Compared with pig and cattle, Paneth cells in sheep attract less attention. In 2003, Ergun et al. comprehensively investigated Paneth cells in ovine small intestine [63]. Ovine columnar Paneth cells are located at crypt bottom and possess abundant acidophilic apical granules. Although Paneth cell number is largest in the ileum of human or mouse, ovine Paneth cells mainly reside in jejunum rather than duodenum and ileum as observed by light microscope [63]. In addition, TEM has revealed that the apical granules in ovine Paneth cells are different in electron densities [63]. High sheep β-defensin-2 (SBD-2) expression is observed in the jejunum and ileum of adult sheep and neonatal lambs, whereas SBD-2 is mainly expressed in the jejunum and colon of pre-term lambs [64]. Although ovine small intestinal organoids have been established for several years [51], the identification of Paneth cells in ovine small intestinal organoids has not been conducted.

SBD-2 staining has revealed that positive regions are mainly located at ovine crypt cells which exhibit similar location and morphology to human and murine Paneth cells. Besides, the expression of SBD-2 is gradually decreased from jejunum to rectum in adult sheep, which could be attributed to the corresponding tissue distribution of ovine Paneth cells [63, 64]. An artificial placenta can improve the care of extremely premature ovine newborns [65]. The application of artificial placenta to premature lambs significantly increases the Paneth cell number in proximal jejunum, suggesting that the increased Paneth cells may play a beneficial role during artificial placenta application [66].

### Paneth cells in horse

In 1994, Kaup and Deegen first identified the presence of Paneth cells in the bottom of equine small intestinal crypts via TB staining and TEM [67]. Equine Paneth cells are also columnar and can be labeled by HE staining, lysozyme staining, and wheat germ agglutinin (WGA) lectin staining [68]. In human or murine small intestine, MUC2 is a goblet cell marker, and UEA1 is used to identify both goblet and Paneth cells [69]. In equine small intestine, MUC2 can label both goblet cells and Paneth cells, but UEA1 staining only visualizes goblet cells. Therefore, MUC2^+^UEA1^−^ is a new labeled pattern of equine Paneth cells [70]. The TEM results show that there seems to be two subtypes of Paneth cells in equine small intestine. Type 1 Paneth cells possess abundant secretory granules and Golgi apparatus, whereas type 2 Paneth cells exhibit a reduction in the secretory granules and the existence of electron-lucent materials, and these two types may represent a young and an old Paneth cell population, respectively [68]. Notably, the number of Paneth cell granules is larger in horse than in other species such as human and mouse, suggesting the important role of equine Paneth cells in maintaining small intestinal homeostasis [67]. In horses, the presence of Paneth cells is only observed in small intestine, and Paneth cell number is smaller in duodenum than that in jejunum or ileum [71].

In equine small intestine, 38 α-defensin transcripts were identified in 2009, and at least 20 α-defensin transcripts could be translated into functional peptides [72]. Equine α-defensin 1 (EAD-1) is only expressed in small intestine. The comparison of the amino acid sequences between EAD-1 and other α-defensin has revealed that EAD-1 is homologous to human α-defensin 5 and rat Paneth cell-derived α-defensin 5 [73]. EAD-1 displays robust antibacterial activity against different Gram-positive and Gram-negative bacteria, as well as horse pathogens mainly including *Rhodococcus equi*, *Salmonella choleraesuis*, and *Pasteurella multocida* [73, 74]. Surprisingly, low concentration EAD-1 (1.25–2.5 μg/mL) has a antibacterial activity similar to conventionally used antibiotics such as ceftiofur and doxycycline, which is observed by the comparison of lethal dose 90% (LD_90_) [74, 75]. Various phospholipids can be aggregated by EAD-1, which may be responsible for its antibacterial ability [76]. The presence of Paneth cells in equine small intestine and the abundant secretory granules in equine Paneth cells indicate that horse is an excellent model animal for the investigation of α-defensins. In addition to α-defensins, lysozyme has also been proved to exist in equine Paneth cells [77]. The expression of Paneth cell marker genes *Lyz* and *Sox9* is detected in equine jejunal organoids [78]. However, *Lyz* expression in organoid-derived monolayers is lower than that in jejunal organoids [78]. This reduction of *Lyz* expression may be attributed to the supplementation of Wnt3a and R-spondin (a Wnt signaling agonist) in monolayer growth medium since the increased Wnt factors lead to a decrease in Paneth cell number in murine intestinal organoid-derived monolayers [79].

Equine AMPs seem to be vital for intestinal immunity. During acute laminitis, the degranulation, a process mediating the release of granules, of Paneth cells is observed in the crypts of whole small intestine (duodenum, jejunum and ileum) [77]. Notably, lysozyme level is high in crypt lumen and the degranulated Paneth cells, suggesting the importance of lysozyme in maintaining small intestinal homeostasis during acute laminitis [77].

Compared with human or murine Paneth cells, equine Paneth cells possess more secretory granules and exhibit high α-defensin content, suggesting that horse could be an appropriate livestock species for the studies of α-defensins. However, the high cost of horse experiment may be a limiting factor.

### Paneth cells in chicken

Chicken Paneth cells are identified based on the presence of lysozyme. In 1974, Humphrey and Turk observed chicken Paneth cells by light-microscopic methods [80]. However, Nile et al. failed to detect the expression of lysozyme *c*, a gene encoding Paneth cell-derived lysozyme, in the small intestine of 17- and 38-day-old chickens, suggesting that there was no Paneth cell in chicken small intestine [81]. This conclusion was also confirmed by the result that the lysozyme positive region was limited in villus but not crypts. The controversy over the presence of chicken Paneth cells is ended until the report that the expressions of lysozyme *c*, lysozyme *g*, and lysozyme *g2* in the crypts of chicken small intestine are detected by quantitative real-time PCR (qPCR) and in situ hybridization [82]. Subsequent PT staining and TEM further confirm the presence of Paneth cells in chicken small intestine. Notably, the expression of lysozyme is not restricted to chicken Paneth cells since lysozyme staining shows the positive regions in both villus and crypts [83]. Recent studies on intestinal organoids and organoid-derived monolayers have also displayed lysozyme expression in chicken small intestinal epithelium [84, 85]. As for morphology, unlike human and murine Paneth cells, chicken Paneth cells are elongated into rod shapes [82]. In addition to lysozyme, β-defensin and angiogenin-4 (ANG4) also exist in chicken Paneth cells [86, 87], but they attract less attention, compared to lysozyme. Serving as a member of ribonuclease A superfamily and another identified AMP, ANG4 has been reported to be secreted by Paneth cells in mice [88].

As a particular intestinal epithelial cell group, Paneth cells are susceptible to intestinal inflammation in humans and mice [12, 89]. Similarly, after LPS treatment, large amount of swollen mitochondria and the reduced lysozyme expression are observed in chicken Paneth cells, suggesting that LPS treatment impairs the homeostasis of Paneth cells [90]. The expression of Paneth cell-derived ISC-supporting factors *Wnt3a* and *Dll1* is decreased at 1 h after LPS challenge, and then gradually increased, which is consistent with the trend of ISC markers *Lgr5* and *Bmi1* expression, suggesting that Paneth cell-derived ISC-supporting factors are responsible for the recovery of ISCs after LPS challenge [90]. Chicken Paneth cells can be also affected by the diets. Wang et al. have established an excellent chicken intestinal organoid culture method, and they have found that methionine deficiency and methionine hydroxy analogue substitution inhibit the growth and development of chicken intestinal organoids and slightly upregulate lysozyme expression [91]. In 2022, Elad Tako from Cornell university demonstrated that Paneth cell number and diameter in chicken duodenum were significantly increased after the separate supplementation of several nutrients including black corn soluble extract, empire apple (juice, pomace and pulp), quinoa soluble fiber, quercetin, and saffron flower water extract [92–96]. In addition, supplementation of organic acids and botanicals down-regulates lysozyme expression in chicken ileum [97], which may be attributed to the antimicrobial activities of organic acids and botanicals since luminal bacteria can stimulate the continuous secretion of lysozyme granules [19]. Probiotics *Lactobacillus salivarius* and *Lactobacillus agilis* can elevate Paneth cell number and *Wnt3a* and *Dll1* expression in duodenum, thus promoting ISC development in chicken [98]. These studies indicate that chicken Paneth cells are sensitive to inflammation, nutrients and bacteria, which is similar to human and murine Paneth cells.

### Paneth cells in rabbit

Rabbit Paneth cells in small intestinal crypts and organoids can be visualized by multiple staining methods such as HE staining, PAS staining, and Masson’s trichrome staining [99, 100]. Rabbit Paneth cells can be also labeled by lectin staining which includes soybean agglutinin (SBA) staining, *Dolichos biflorus* agglutinin (DBA) staining, and WGA staining [99]. Notably, the shape of rabbit Paneth cells tends to be circular [99]. However, rabbit Paneth cells are not observed in intestinal organoid-derived monolayers [101]. Although lysozyme has been considered as a Paneth cell marker in various animal species, lysozyme staining fails to label Paneth cells in rabbit small intestinal crypts [99, 101]. Future work should be conducted to investigate whether rabbit Paneth cells contain lysozyme and whether rabbit lysozyme reacts with commercial lysozyme antibody. The number of rabbit Paneth cells exhibits a gradual increase from the duodenum to the ileum [100]. Additionally, the TEM results have shown that Paneth cells are present in the small intestine of hare [102].

Although whether rabbit Paneth cells contain AMPs remains unclear, glucose-6-phosphate dehydrogenase (G6PD) has been reported to be expressed in rabbit Paneth cells. Large amount of G6PD protein is located at the rough endoplasmic reticulum and Golgi network in rabbit Paneth cells [103]. G6PD can provide NAPDH for multiple biological processes [104–106]. Thus, it is possible that G6PD in Paneth cells could produce NAPDH to fuel the reduction of thiols of cryptdins [107]. The degranulation process of Paneth cells can be triggered by toll-like receptor 9 (TLR9) activation in mice [108]. *Lactobacillus casei* administration induces the upregulated expression of TLR9, defensin-7-like, and lysozyme, as well as the degranulation process of Paneth cells in rabbit duodenum and jejunum [109].

### Paneth cells in other farm animal species

In contrast to the farm animal species mentioned above, studies of Paneth cells in other species are relatively rare. The expression of lysozyme is detected in the jejunum of ducks, and zinc supplementation elevates the level of lysozyme expression [110]. However, duck Paneth cells have been not identified via staining so far. TB staining and Masson’s trichrome staining exhibit positive region in the secretory granules of cells in goose small intestinal crypts, suggesting the presence of Paneth cells in geese [111]. Additionally, there are abundant mitochondria and rough endoplasmic reticula in goose Paneth cells, which is similar to human and murine Paneth cells [111]. Masson’s trichrome staining results have indicated that *Toxoplasma gondii* infection leads to a significant increase in Paneth cell number in cat duodenum [112].

## Future directions for research on Paneth cells in farm animals

Current research on Paneth cells in the reviewed animals mainly focuses on Paneth cell identification and Paneth cell-derived AMP examination (Table 1). However, compared with the knowledge on human and murine Paneth cells, our understanding of Paneth cells in farm animals is relatively poor. The efforts to explore the detailed information such as AMP composition and Paneth cell function are required for the comprehensive understanding of Paneth cells in farm animals.

**Table 1:** The identified composition of Paneth cell granules in farm animals

Farm animal	Proteins in granules	Methods	References
Pig	Lysozyme	IHC	[35]
		scRNA-seq	[40]
		IF	[33, 37]
Cattle	Lysozyme	mRNA-seq	[53]
	β-defensins	mRNA-seq	[53]
	ITLN2	IHC	[61]
	LBP	qPCR	[58]
Sheep	SBD-2	IHC	[64]
Horse	Lysozyme	IHC	[68, 77]
		qPCR	[78]
	EAD-1	qPCR	[73]
Chicken	Lysozyme	IHC	[83]
		IF	[84]
		qPCR	[85]
		WB	[85]
	β-defensin	IHC	[87]
	ANG4	qPCR	[86]
Rabbit	G6PD	IHC	[104]

*ANG4* Angiogenin-4, *EAD-1* Equine α-defensin 1, *G6PD* Glucose-6-phosphate dehydrogenase, *IF* Immunofluorescence, *IHC* Immunohistochemistry, *ITLN2* Intelectin 2, *LBP* Lipopolysaccharide-binding protein, *qPCR* Quantitative real-time PCR, *SBD-2* Sheep β-defeinsin-2, *scRNA-seq* Single-cell mRNA sequencing, *WB* Western blot

Although scRNA-seq has confirmed the presence of porcine Paneth cells, staining results for Paneth cell identification are still inconsistent in pigs. Therefore, developing a mature method to stain porcine Paneth cells is urgently required for the investigation on porcine Paneth cells.

In mice and rats, gene editing and dithizone (a metal chelator) have been used to disrupt Paneth cells in small intestine, making it feasible to explore Paneth cell function [14, 113–115]. Due to its high cost and complexity, gene editing is infrequently applied to livestock. Dithizone is utilized to establish a Paneth-cell-disruption model in mice and rats [14, 113]. It is worthwhile to investigate whether dithizone administration could deplete Paneth cells in the small intestine of different livestock species, which is likely to provide an excellent Paneth-cell-disruption model for subsequent research on Paneth cells in farm animals. Intestinal microbiota plays an important role in nutrient processing and intestinal immunity [116], and ISCs are responsible for the renewal and regeneration of intestinal epithelium [117]. Considering microbiota-regulating and ISC-supporting roles of Paneth cells, the maintenance of functional Paneth cells is of importance for intestinal homeostasis [18]. Under the condition of intestinal disorders such as PEDV infection and TGEV infection, Paneth cells and AMPs are generally impaired, thus leading to the weakened microbiota control and ISC activity [35, 50]. In recent years, many above-mentioned nutrients or ingredients in this review have been reported to have the ability to improve Paneth cells in farm animals under intestinal homeostasis or disorders [41, 95]. Therefore, developing proper nutritional regulation techniques is a feasible strategy to protect the functional Paneth cells in farm animals, thus improving the intestinal health.

In mice, the recovery of functional Paneth cells through α-defensin or lysozyme supplementation can effectively alleviate intestinal injury caused by dextran sulfate sodium (DSS) treatment, *Helicobacter hepaticus* infection, or acute pancreatitis [13, 118–120]. Notably, lysozyme treatment increases the number of Paneth cells in murine small intestinal organoids [119]. In pigs, lysozyme supplementation alleviates intestinal injury and inflammation in a malnutrition and enterotoxigenic *Escherichia coli* infection model [121]. In chickens, exogenous lysozyme supplementation inhibits the overgrowth of *Escherichia coli* and *Lactobacillus* and reduces the intestinal lesion scores after *Clostridium perfringens* infection [122]. *Clostridium perfringens* infection induces the overgrowth of *Lactobacillus,* which further contributes to resisting *Clostridium perfringens* infection. Exogenous lysozyme supplementation may enhance intestinal immunity to directly resist *Clostridium perfringens* infection in chickens, which reduces the dependence on *Lactobacillus* to resist *Clostridium perfringens* infection. Thus, future work could develop novel AMP products and utilize them to alleviate intestinal injury and inflammation in livestock.

Although it is generally accepted that Paneth cells devote to maintaining intestinal homeostasis, Paneth cells are detrimental to intestinal health in some cases. Abnormal Paneth cells can trigger intense intestinal inflammation in mice [89]. It has been reported that Paneth cell-derived IL-17A is responsible for the injury and inflammation in intestine, liver and kidney during ischemia reperfusion, and the neutralization of IL-17A effectively alleviates organ injury and inflammation [123, 124]. Abundant IL-1β is expressed in Paneth cells under intestinal homeostasis, and Paneth cell-derived IL-1β causes severe damage to intestinal epithelial barrier in simian immunodeficiency virus infection [125]. However, there has been no report on the pro-inflammatory cytokine in Paneth cells in farm animals. It is worth examining whether there are some pro-inflammatory cytokines present in Paneth cells of the reviewed animals or not via immunostaining, fluorescence in situ hybridization, or flow cytometry during intestinal disorders, thus developing some targeted strategies.

**Fig. 3:**
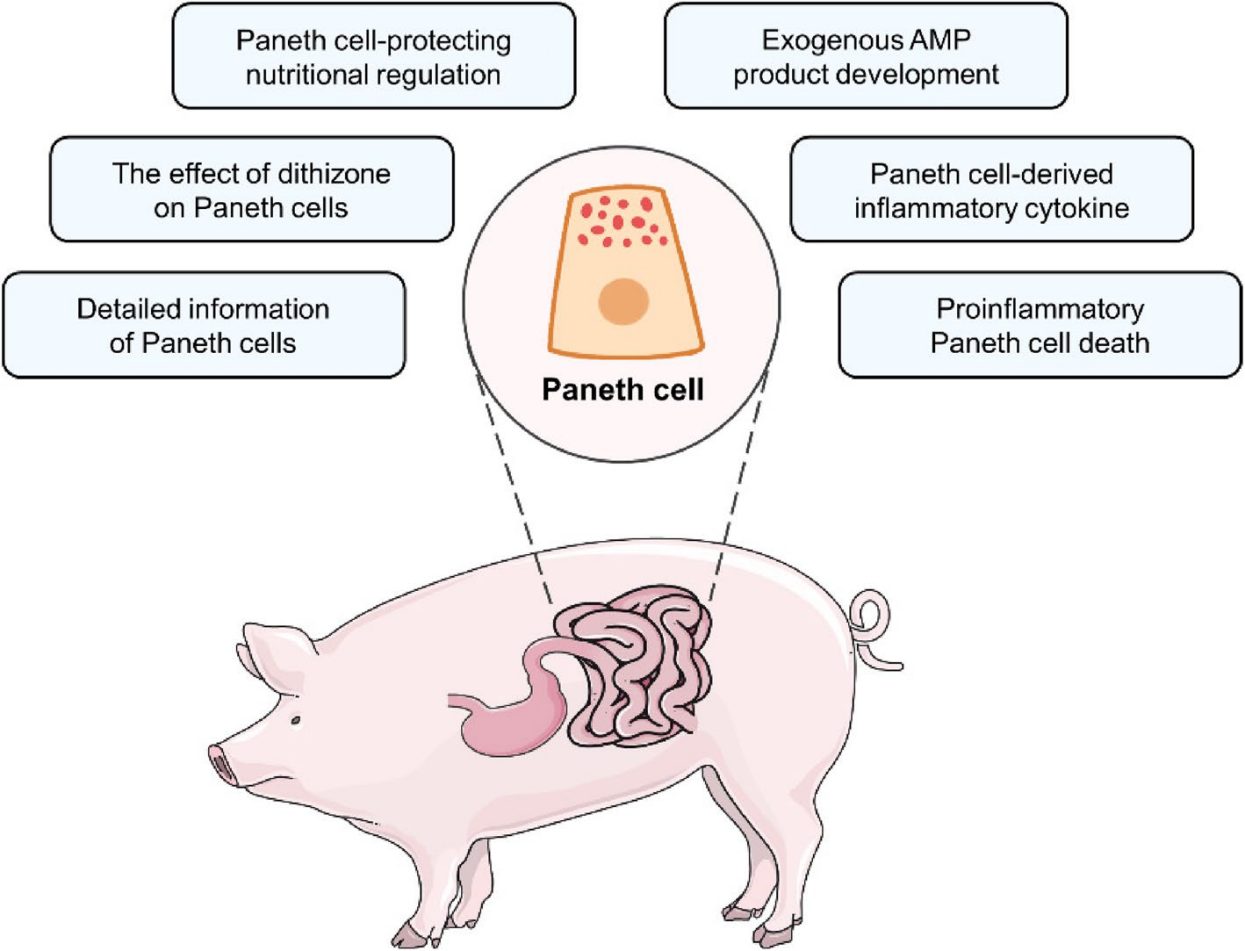
Future directions for the investigation of farm animal Paneth cells mainly include six aspects: the investigation of more detailed information of Paneth cells, the effect of dithizone on Paneth cells, the development of Paneth cell-protecting nutritional regulation, the development of exogenous AMP products, the examination of Paneth cell-derived inflammatory cytokine, and the investigation of proinflammatory Paneth cell death

Compared with Paneth cell differentiation and functions, Paneth cell death attracts less attention. Necroptosis, a particular form of programmed cell death, has been observed in Paneth cells of the inflamed human and murine small intestine [12]. Paneth cells are susceptible to necroptosis due to the high expression of receptor-interacting protein 3 (RIP3), a key protein of necroptosis [12]. Necroptotic cells can release many immuno-stimulating damage-associated molecular patterns (DAMPs) such as mitochondrial DNA and ATP, thus triggering the immune reaction of immune cells [126]. After LPS challenge, the elevated expression of RIP1 and high mobility group box 1 (HMGB1), two key necroptosis proteins, is detected in the porcine jejunal crypts where Paneth cells are located [127]. However, this study cannot demonstrate the presence of necroptotic porcine Paneth cells during LPS challenge since the necroptotic cells in porcine jejunal crypts might be not Paneth cells. Future work should investigate whether Paneth cells in farm animals could undergo necroptosis during intestinal inflammation and the role of potential necroptotic Paneth cells in intestinal inflammation (Fig. 3).

## Conclusion

Paneth cells serve as the guardians of small intestine due to their multiple functions. Although the existence of Paneth cells in pigs and chickens had once been controversial, recent studies have confirmed that Paneth cells are present in the small intestine of most farm animals, as demonstrated by various staining methods. In addition to AMPs, there are also some functional proteins existing in Paneth cells in farm animals, such as LBP and G6PD. Paneth cells in different livestock species are susceptible to intestinal disorders and diets since these factors significantly affect Paneth cell number or AMP expression in farm animals. Paneth cell identification and Paneth cell-derived AMP examination in farm animals are far behind the studies of human or murine Paneth cells. In this review, we first summarize the current research status of Paneth cells in farm animals and provide future directions for research on Paneth cells in farm animals. Future work is urgently needed to investigate Paneth cell function and develop Paneth cell-based intervention strategies against intestinal disorders in livestock, thus promoting the healthy development of husbandry.

## Data Availability

Not applicable.

## Abbreviations

ADFI: Average daily feed intake
ADG: Average daily gain
AMP: Antimicrobial peptide
ANG4: Angiogenin-4
CD: Crohn's disease
DAMPs: Damage-associated molecular patterns
DBA: *Dolichos biflorus* agglutinin
DON: Deoxynivalenol
DSS: Dextran sulfate sodium
EAD-1: Equine α-defensin 1
EGF: Epidermal growth factor
G6PD: Glucose-6-phosphate dehydrogenase
HE: Hematoxylin–eosin
HMGB1: High mobility group box 1
IF: Immunofluorescence
IHC: Immunohistochemistry
IgA: Immunoglobulin A
ISCs: Intestinal stem cells
ITLN2: Intelectin 2
LBP: Lipopolysaccharide-binding protein
LD_90_: Lethal dose 90%
LPS: Lipopolysaccharide
NEC: Necrotizing enterocolitis
PAS: Periodic acid-Schiff
PCNA: Proliferating cell nuclear antige
PDCoV: Porcine deltacoronavirus
PEDV: Porcine epidemic diarrhea virus
PT: Phloxine tartrazine
PTB: Paratuberculosis
qPCR: Quantitative real-time PCR
RIP3: Receptor-interacting protein 3
ROC: Receiver operating characteristic
ROS: Reactive oxygen species
SBA: Soybean agglutinin
SBD-2: Sheep β-defeinsin-2
scRNA-seq: Single-cell mRNA sequencing
Sox9: Sex determining region Y-box 9
TB: Toluidine blue
TEM: Transmission electron microscopy
TGEV: Transmissible gastroenteritis virus
TLR9: Toll-like receptor 9
WB: Western blot
WGA: Wheat germ agglutinin
